# Nitrosative Stress in Astronaut Skeletal Muscle in Spaceflight

**DOI:** 10.3390/antiox13040432

**Published:** 2024-04-02

**Authors:** Dieter Blottner, Manuela Moriggi, Gabor Trautmann, Sandra Furlan, Katharina Block, Martina Gutsmann, Enrica Torretta, Pietro Barbacini, Daniele Capitanio, Joern Rittweger, Ulrich Limper, Pompeo Volpe, Cecilia Gelfi, Michele Salanova

**Affiliations:** 1 Institute of Integrative Neuroanatomy, Charité—Universitätsmedizin Berlin, Corporate Member of Freie Universität Berlin, Humboldt-Universität zu Berlin, and Berlin Institute of Health, 10115 Berlin, Germany; gabor.trautmann@charite.de (G.T.); katharina.block@charite.de (K.B.); martina.gutsmann@charite.de (M.G.); michele.salanova@charite.de (M.S.); 2 NeuroMuscular System and Signaling Group, Center of Space Medicine and Extreme Environments, 10115 Berlin, Germany; 3 Department of Biomedical Sciences for Health, University of Milan, 20133 Milan, Italy; manuela.moriggi@unimi.it (M.M.); pietro.barbacini@unimi.it (P.B.); daniele.capitanio@unimi.it (D.C.); cecilia.gelfi@unimi.it (C.G.); 4 C.N.R. Neuroscience Institute, I-35121 Padova, Italy; sfurlan@mail.bio.unipd.it; 5 Laboratory of Proteomics and Lipidomics, IRCCS Orthopedic Institute Galeazzi, Via R. Galeazzi 4, 20161 Milan, Italy; enrica.torretta@grupposandonato.it; 6 Institute of Aerospace Medicine, German Aerospace Center (DLR), 51147 Cologne, Germany; joern.rittweger@dlr.de (J.R.); ulrich.limper@dlr.de (U.L.); 7 Anesthesiology and Intensive Care Medicine, Merheim Medical Center, Witten/Herdecke University, 51109 Cologne, Germany; 8 Department of Biomedical Sciences, Università di Padova, I-35121 Padova, Italy; pompeo.volpe@unipd.it

**Keywords:** spaceflight, nitric oxide synthase, microgravity, RONS, nitrosated proteins

## Abstract

Long-duration mission (LDM) astronauts from the International Space Station (ISS) (>180 ISS days) revealed a close-to-normal sarcolemmal nitric oxide synthase type-1 (NOS1) immunoexpression in myofibers together with biochemical and quantitative qPCR changes in deep calf soleus muscle. Nitro-DIGE analyses identified functional proteins (structural, metabolic, mitochondrial) that were over-nitrosylated post- vs. preflight. In a short-duration mission (SDM) astronaut (9 ISS days), s-nitrosylation of a nodal protein of the glycolytic flux, specific proteins in tricarboxylic acid (TCA) cycle, respiratory chain, and over-nitrosylation of creatine kinase M-types as signs of impaired ATP production and muscle contraction proteins were seen. S-nitrosylation of serotransferrin (TF) or carbonic anhydrase 3 (CA3b and 3c) represented signs of acute response microgravity muscle maladaptation. LDM nitrosoprofiles reflected recovery of mitochondrial activity, contraction proteins, and iron transporter TF as signs of muscle adaptation to microgravity. Nitrosated antioxidant proteins, alcohol dehydrogenase 5/S-nitrosoglutathione reductase (ADH5/GSNOR), and selenoprotein thioredoxin reductase 1 (TXNRD1) levels indicated signs of altered redox homeostasis and reduced protection from nitrosative stress in spaceflight. This work presents a novel spaceflight-generated dataset on s-nitrosylated muscle protein signatures from astronauts that helps both to better understand the structural and molecular networks associated to muscular nitrosative stress and to design countermeasures to dysfunction and impaired performance control in human spaceflight missions.

## 1. Introduction

In human space life sciences, the typical (and still not fully understood) loss of muscle mass and function in astronauts was recently highlighted by new research priorities. These priorities focus on fundamental molecular mechanisms of space-related musculoskeletal disuse and adaptation [1], muscle and bone loss [2], nutrition and metabolism [3], and the need for optimization of exercise as countermeasures (CMs), as exemplified by the targeting of certain muscle groups of the human body as prime “executers” of motion in Earth gravity (1G) or variable gravity (ΔG) vectors (e.g., Moon, Mars) in future deep space explorations [4,5,6]. 

Changes in redox genes and signal-related pathways are signatures of long-term exposure of astronauts to extreme space conditions [7] with as-yet unknown crew health risks. Attempts to trigger exercise-induced antioxidant responses in normal muscle [8] or by therapeutical denitrosylation in various human diseases [9] are promising, as are new strategies to combat antioxidant capacity, such as by exercise-driven interventions in normal conditions [10], chronic disuse in bed rest (a ground-based analogue to spaceflight [11]), and dietary nutritional intervention [12]. Taken together, these approaches inspired the hope for a better management of oxidative/nitrosative stress in normal and clinical populations but also for astronaut musculoskeletal health, in particular during long-duration mission (LDM) spaceflights (>6 months or more).

Reactive oxygen (ROS) and nitrogen species (RNS) induced by free radicals (e.g., ONOO peroxides), collectively termed ‘reactive oxidative/nitrosative species’ (RONS) [13,14], are known common stressors in disuse atrophy [15] and inter-organ communication [16], as, for example, seen in muscle wasting diseases and aging [17], that result from imbalanced production or expression in cells and tissues under various conditions. For example, nitrosative stress in dystrophic muscle disease [18] or in chronic disuse conditions with otherwise healthy volunteers in bed rest is characterized by an imbalance of nitric oxide synthase (NOS) expression and regulation, promoting mutable free radical NO signals associated to s-nitrosylation (SNO) of functional muscle proteins [19,20]. Moreover, three major sources of the biologic gaseous NO signals were found in brain neuronal cells (via neuronal NOS, nNOS/NOS1), immune system (macrophages, inducible NOS2), and vascular system (endothelial cells, endothelial NOS3), but also in skeletal muscle (NOS1, µNOS) [21].

Skeletal muscle (taken together, forming the largest body organ) is a major source of NO in the human body [22]. NO production is maintained via both the NOS-dependent pathway (e.g., L-arginine + O_2_ -> NO) and the NOS-independent pathways (e.g., NO_3_ -> NO_2_ -> NO, -> mostly via leafy vegetable diet [23]). In muscle physiology, NO serves as a critical molecular signal during development, growth [24,25,26,27], neuromuscular formation [28], muscle contraction [29], microcirculation [30], and exercise tolerance [27]. In exercise physiology, dietary nitrate supplementation (e.g., sodium nitrate [NaNO_3_], NO_3_-rich beetroot juice) has been highlighted as potential nutritional “ergogenic aids” to promote ‘physical performance for health’ benefits [23]. In muscle pathophysiology, NO is involved in muscle repair [31], wound healing and inflammation [32], impaired muscle perfusion [33], and microcirculation [34] as well as in post-exercise muscle cramps [35] and in various muscle weakness diseases [36]. 

Robust inactivity-induced NOS changes in murine skeletal muscle have been demonstrated both in ground-based studies (tissue sharing) and in space-flown mice on the NASA/ASI Mouse Drawer System (MDS) mission (91 days on ISS, NASA STS-129), thus confirming microgravity-induced delocalization of membrane/cytosolic NOS1 immunosignals in postural murine hindlimb skeletal muscle [37]. A previous bed rest study has shown extensive NOS1 immunosignals at the skeletal muscle–myofiber outer membranes of the soleus muscle, suggesting robust NOS re-localization following resistive exercise as a countermeasure in otherwise healthy musculature [38]. In another bed rest study, we have demonstrated targeted SNO levels of functional proteins such as nitrosylated ryanodine receptor type 1 (SNO-RyR1) and several others involved in contraction coupling and related proteins that clearly showed aberrant SNO protein abundance as signatures of immobilization-induced muscle disuse attenuated by resistive vibration exercise [19]. We thus hypothesized that NOS/NO signalling and key nitrosative stress indices via RONS products are linked both to muscle activity/inactivity and to microgravity-unloaded skeletal muscle. If so, SNO signatures in skeletal muscle proteins could then serve as biomarkers and thus be reflecting efficacy of inflight exercise protocols as countermeasures (CMs) to offset or mitigate sarcolemmal NOS dislocation in myofibers and aberrant SNO patterns of key muscle proteins. Investigation of SNO post-translational protein modifications in long-term spaceflight helps to shed light on RONS-associated and space-related disuse atrophy based on disrupted redox homeostasis in skeletal muscle. 

In the present study, we report novel results from the MUSCLE BIOPSY (ESA op.nom.) experiment onboard the International Space Station (ISS) using pre- and postflight biopsy tissue samples available from LDM astronauts’ lower leg (calf) soleus muscle [39]. Our first study aim was to compare sarcolemmal NOS1 immuno-localization patterns directly in soleus muscle samples from a short-duration mission (SDM) astronaut (9 days on ISS, without inflight exercise) with those available from LDM astronauts (>6 month or more on ISS, with routine inflight exercise). In particular, pixel-intensity analyses of sarcolemmal NOS1 immuno-expression patterns were used as signs of a potential NO source at the muscle fiber sub-cellular level. Our second study aim was to investigate differential changes in SNO protein levels using small amounts of soleus muscle biopsy lysates from astronauts subjected to un-targeted Nitro-DIGE analysis [40]. In addition to global nitroso-profiles of canonical proteoforms involved in energy metabolism, transport, stress response, and muscle contraction, we were interested in more specific SNO signatures in disused skeletal muscle from two of the most prominent complex enzyme systems triggered by the Nuclear factor (erythroid-derived 2)-2 (Nrf2), such as the thyoredoxin (TRX) and S-Nitrosoglutatione reductase (GSNOR) systems, which are necessary for the maintenance of protein s-nitrosylation/denitrosylation homeostasis [41]. 

## 2. Materials and Methods

### 2.1. Study Participants 

The present work (op. nom. MUSCLE BIOPSY; European Space Agency (ESA)’s Science & Exploration Research Plan, ILSRA-2004-155) includes data from six (*n* = 6 valid) male ISS crew members enrolled from the United States Orbital Segment (USOS) astronaut corps from the National Aeronautics and Space Agency (NASA), the Japanese Space Exploration Agency (JAXA), and the European Space Agency (ESA). One study participant, astronaut A, was assigned to a short-duration mission (SDM) (9 days on ISS), and five other astronauts, B to F (*n* = 5), were assigned to a long-duration mission (LDM, ~180 days/6 months on ISS). Anthropometric data (body mass indices), general study protocol, and total numbers of inflight physical exercise countermeasure (CM) days (walking/running on T2, cycling with CEVIS, weight lifting with aRED) for the participating astronauts are published elsewhere [39]. 

### 2.2. Muscle Biopsy

From the study participants of MUSCLE BIOPSY, we collected one pre-flight (L-90 ± 30 days prior to launch) and one post-flight biopsy (return day R+0/1, landing day) from the right soleus muscle, a major slow-type and postural deep calf skeletal muscle, by an established protocol procedure either at the European Astronaut Center (EAC)/Human Physiology Lab at the DLR Institute of Aerospace Medicine, German Aerospace Center, Deutsches Zentrum für Luft- und Raumfahrt (DLR:envihab, Cologne, Germany), or at NASA’s Johnson Space Center (JSC, Houston, TX, USA), depending on the astronaut’s return travel schedules, as previously reported [39]. Biopsy material was immediately frozen in liquid nitrogen on site and stored at minus 80 °C in a freezer until further use.

### 2.3. Immunohistochemistry

Serially cut cryosections (8–10 µm thickness, obtained by using a cryostat CM-1860, leica-microsystems.com, accessed on 21 March 2024) from frozen tissue blocks were mounted on glass slides (Superfrost, Thermo Fisher Scientific, Henningsdorf, Germany) and stored frozen (−80 °C) in sealed boxes until further use for analyses together with an established immunostaining protocol in parallel to avoid any bias in laboratory staining outcome across the study duration. Cryosections were fixed (4% buffered paraformaldehyde, 10 min, 4 °C), rinsed in physiological PBS buffer, and preincubated with mouse IgG blocking reagent (1:500 M.O.M., Vector Laboratories Inc., Newark, CA, USA) in buffer. We used monoclonal antibodies raised against nitric oxide synthase type-1 (anti-NOS, monoclonal, sc-5302, N-terminal amino acid 2-300, Santa Cruz Biotechnology, Dallas, TX, USA). Primary antibodies were visualized by using fluorochrome Alexa-488 and Alexa-555 conjugated secondary antibodies (affinity purified goat-anti-mouse IgG, Invitrogen, Waltham, MA, USA) using an established experimental protocol [38]. Confocal imaging was performed with a high-resolution three-channel laser confocal microscope (SP-8, leica-microsystems.com, accessed on 21 March 2024). For quantification of subcellular immunofluorescence signal intensities (pixel intensity per µm^3^ and image volume given as arbitrary units, A.U.), each cryosection was subdivided into several non-overlapping regions of interest (ROIs), thus covering most of the cross-sectioned muscle fiber profiles identified with a sarcolemma biomarker (1:1000, monoclonal anti-dystrophin, NCL-DYS2 #107416, Novocastra Antibodies, Newcastle, UK) and with a myonuclear biomarker 4’,6-diamidino-2-phenylindole fluorescent DNA stain (1:1000, DAPI, Hoechst 33258 stain), and inspected with a 40× objective (SP8 laser scanning microscope, leica-microsystems.com, accessed on 21 March 2024). The number of fields (ROIs), however, varied between 4 and 16 fields among cryosections due to the size of the cross-sectional planes from each biopsy sample.

### 2.4. Proteomics with Nitro-DIGE

#### 2.4.1. Protein Extraction

Muscle biopsies taken before and after the flight were suspended, sonicated, clarified, and quantified as described previously [39]. 

#### 2.4.2. Identification of S-Nitrosated Proteins by 2-D CyDye-Maleimide DIGE (Nitro-DIGE)

A modified biotin switch method was used to identify SNO proteins, as previously described [42]. For each sample, two technical replicates were considered and labeled with Cy5, while a mixture containing an equal amount of all samples was labeled with Cy3 as the internal standard.

#### 2.4.3. Image Acquisition 

Images from CyDye-labeled gels were acquired by Typhoon 9200 Imager (GE HealthCare, Solingen, Germany), and image analysis was performed by DeCyder software (version 6.5, GE HealthCare). For each experimental group (post- and pre-SDM; post- and pre-LDM), proteins identified in at least 70% of samples were considered. 

#### 2.4.4. Protein Identification

Protein identification was carried out by matrix-assisted laser desorption/ionization time-of-flight (MALDI-ToF) mass spectrometry (MS), as previously described [42].

#### 2.4.5. Immunoblotting

Protein extracts (50 µg) from one SDM (acute exposure) and two LDM astronauts (chronic exposure) were loaded in duplicate and resolved on 10–16% gradient polyacrylamide gels. Blots were incubated with primary antibodies as follows: mouse monoclonal anti-alcohol dehydrogenase 5/S-nitrosoglutathione reductase (ADH5/GSNOR, 40 kDa, Santa Cruz Biotechnology, Dallas, TX, USA, sc-293460, 1:500), mouse monoclonal anti-thioredoxin reductase 1 (TXNRD1, 55 kDa, Santa Cruz Biotechnology, Dallas, TX, USA, sc-28321, 1:500), rabbit polyclonal anti-nitric oxide synthase 1 (NOS1, 155KDa, Santa Cruz Biotechnology, Dallas, TX, USA, sc-8309, 1:500). The membranes were then incubated with anti-mouse secondary antibodies conjugated to horseradish peroxidase (KPL, Seracare, Milford, MA, USA, 1:5000). The ECL Prime Detection Kit and the Image Quant LAS 4000 analysis system (GE Healthcare) were used to visualize the signals by chemiluminescence. 

#### 2.4.6. RNA Extraction and qPCR 

Total RNA was extracted from small tissue pieces of soleus using a commercially available TRIzol Reagent (Thermo Fisher Scientific) following the manufacturer’s instructions. The concentration and purity of the RNA were determined using NanoDrop Lite Spectrophotometer (Thermo Fisher Scientific) by measuring the absorbance at 260 (OD260) and 280 (OD280) nm. Following isolation, 1 μg of RNA was reverse transcribed into cDNA using a VILO cDNA synthesis kit (Thermo Fisher Scientific). Primers for reference genes B2M and PPIA were published elsewhere [42]. Specific primers for NOS1 (NOS1, *Homo sapiens* (human) gene ID:4842) were designed using Primer3 software (http://frodo.wi.mit.edu/, accessed on 1 February 2024, version 4.1.0, Whitehead Institute for Biomedical Research, Camebridge, MA, USA) and their thermodynamic specificity was determined using BLAST sequence alignment (NCBI) and vector NTI^®^ software (Thermo Fisher Scientific). No Genomic DNA interference was allowed because cDNAs of interest were amplified using the pair of primers derived from different exons of the genes. Human NOS1 specific primers:

Fw: CAATGTGCCTGTCGTCCTCA; Rv: GTGCATCCCGTTTCCAATGT

Quantitative PCR was performed using SYBR green chemistry (Bio-Rad Laboratories GmbH, Neuried, Germany) in triplicate in a 96-well CFX Thermal Cycler (Bio-Rad). All samples were run in parallel with RNA- and RT-negative controls. Efficiency of each run was monitored by a standard curve. Normalization was performed by 2^−∆∆Ct^ algorithm to determine the relative fold change of gene expression of samples. B2M and PPIA were tested as putative reference genes, with B2M being the most stable gene to normalize cycle threshold (Ct) values.

### 2.5. Statistics

For NOS1 immunosignal analyses, we used the Pearson–D’Augustino normality test (pre vs. post) for Gaussian normal distribution, together with parametric unpaired *t*-test and Mann–Whitney test (non-Gaussian). Significance level was set at *p* ≤ 0.05, or even higher (*p* ≤ 0.001) as indicated. GraphPad Prism (v9.4.0) was used for graphical representation and statistical data analyses. Due to highly limited amount of tissue available, sarcolemmal NOS1 immunosignal intensities were pooled and determined in at least three different cryosections per each sample with at least 50 cross-sectioned myofibers (type 1 or 2) at two different time points (pre- vs. postflight). Image analysis of immunostained cryosections was performed at identical settings with Leica Application Suite (LAS) X co-localization/3D image analysis software (release #3.5.7.23225.3D, www.leica-microsystems.com, accessed on 21 March 2024). 

For Nitro-DIGE data analysis, statistically significant differences were computed by paired Student’s *t*-test with a *p*-value threshold of 0.05. False discovery rate was applied to correct for multiple tests to reduce the overall error.

For immunoblotting data analysis, band quantification was performed using the Image Quant TL v. 8.1(GE HealthCare) software followed by statistical analysis (Student’s *t*-test, *p*-value ≤ 0.05). Band intensities were normalized against the total amount of proteins stained by Sypro ruby total protein stain.

## 3. Results

### 3.1. Quantitative PCR (qPCR) Analyses of NOS1 Transcripts in Astronaut Soleus Muscle Samples

We analyzed skeletal muscle biopsy samples obtained from short- (SDM) and long-duration mission (LDM) astronauts before and after spaceflight. Astronaut A belongs to a short-duration mission (SDM-A; 11-day spaceflight with 9 ISS days) and was not obliged to regular Medical Operational (MedOps) inflight exercise because of the short flight duration and stay onboard the ISS. In contrast, LDM astronauts (LDM-B, C, D, and E) performed regular inflight exercise as a countermeasure (1.5 to 2 h daily) during their entire mission onboard the ISS.

**Figure 1:**
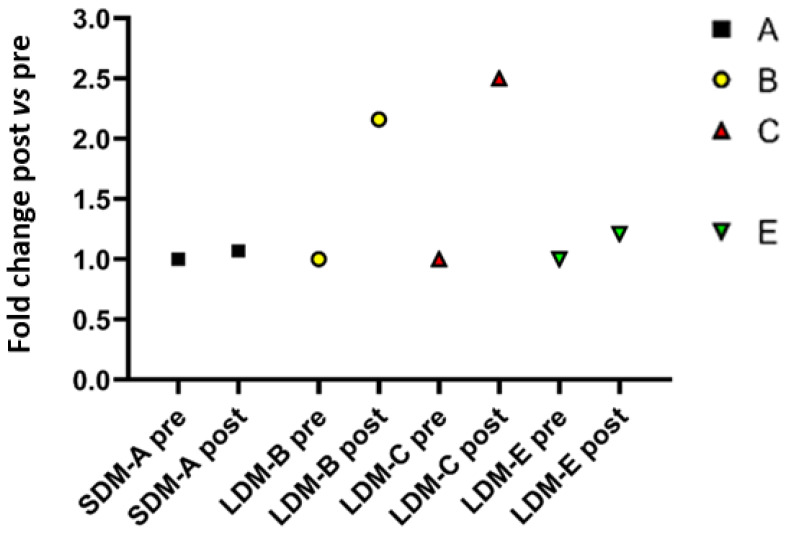
Scatter plot of NOS1 quantitative PCR (qPCR) data from soleus muscles from one (*n* = 1) SDM astronaut (SDM-A, 9 ISS days, black squares) and three (*n* = 3) LDM astronauts (LDM-B, yellow circle/C, red triangles/E, green triangles, 180 ISS days or more). NOS1 transcript levels were elevated in LDM-B and C (2–2.5-fold change) or in LDM-E (trend only) vs. SDM condition post- vs. preflight. Astronaut D values not available (tissue limitation).

As shown in Figure 1, quantitative PCR analysis in soleus muscle samples pre- vs. post-flight showed that in two out of three LDM subjects (LDM-B and C), there was a 2- to 2.5-fold change in NOS1 transcripts that was not seen in SDM-A or LDM-E (trend only).

### 3.2. NOS1 Immunolocalization in Astronaut Soleus Muscle Cryosections

**Figure 2:**
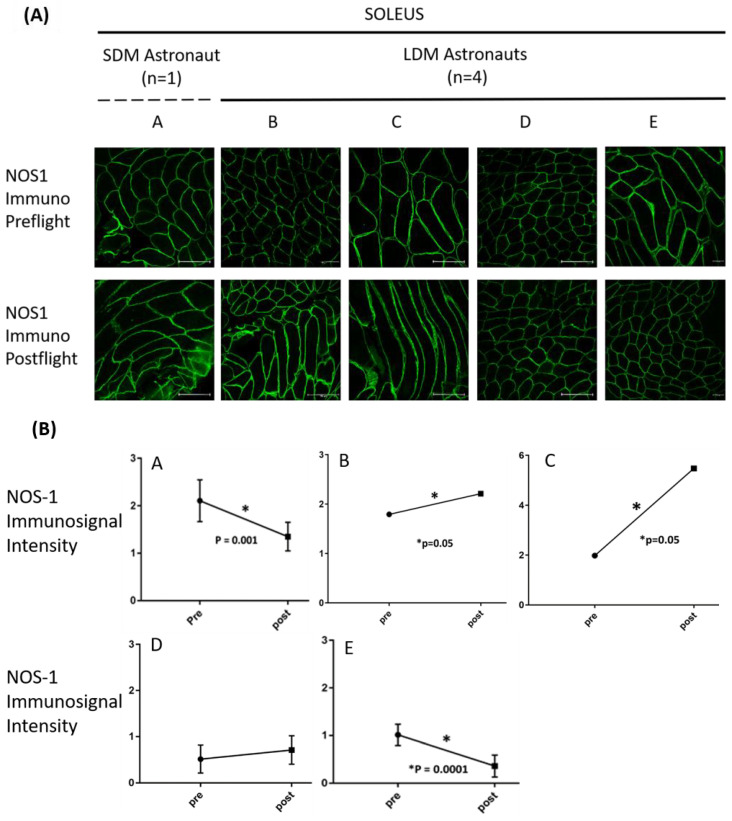
Nitric oxide synthase type-1 (NOS1) subsarcolemma immunoexpression (green fluorescence) in soleus muscles before (pre-flight) and after (post-flight) spaceflight. (**A**) SDM astronaut A is considered as a “non-exercise” control compared to the four LDM astronauts (B to E) with routine inflight exercise as a countermeasure. (**B**) NOS1 immunosignal intensity semi-quantification by three-dimensional (3D) confocal image analysis (mean pixel intensity per NOS1 positive volume/image size, in µm^3^) in astronauts (y-axis: arbitrary units, a.u.) pre-flight (pre) vs. post-flight (post). Bar (images) = 150 µm. * *p*-values (graphs) ranged from significance level *p* = 0.001 to 0.0001.

**Figure 3:**
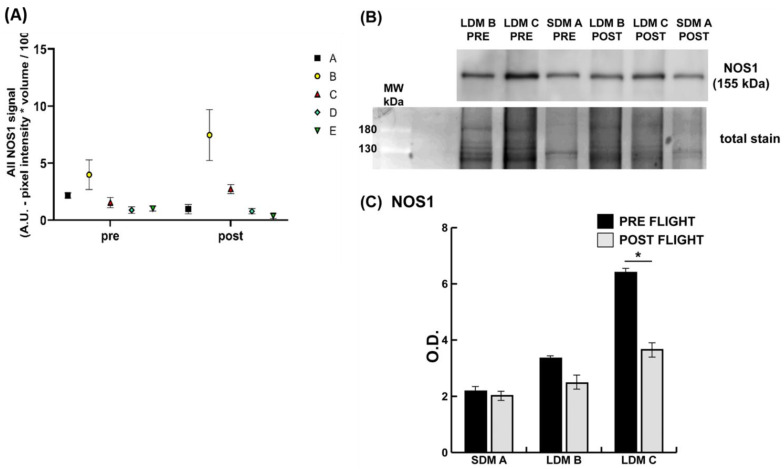
(**A**) Graph (scatter plot) showing sarcolemmal anti-NOS1 immunosignal pixel changes (pre- vs. postflight) in the soleus muscle (left panel) of LDM astronauts B, C, D, and E (*n* = 4) vs. one SDM astronaut (A, *n* = 1) as reference control. Color codes of astronaut values (as presented by square, circle, triangles, diamond symbols) highlight inter-subject variability (see astronaut B and E). (**B**) Representative NOS1 immunoblot images from SDM-A, LDM B, and LDM-C. (**C**) Representative bar graph (means ± SD) showing quantitative pre-flight (black bars) vs. post-flight (grey bars) NOS1 protein change in soleus lysates. Data were normalized against the total amount of loaded proteins stained with Sypro Ruby. O.D. = optical density; * significant difference; Student’s *t*-test, *n* = 2, *p* < 0.05. Full-length images are available in the Supplementary Materials. Astronaut D and E immunoblot values not available (tissue limitation).

Figure 2 and Figure 3A show qualitative and quantitative analysis of subsarcolemma-associated NOS1 immunosignal intensity values in cryosections of the postural deep calf soleus muscle from SDM astronaut A (little or no exercise) before (pre-flight) and after spaceflight (post-flight). In the SDM astronaut, sarcolemma NOS1 immunosignal was reduced (*p* = 0.001) in muscle post-flight shortly (R0/+1) after landing vs. pre-flight (Figure 2 and Figure 3A).

Quantitative analysis of NOS1 immunosignal pixel intensities confirmed that, in the soleus muscle of at least two LDM crew members (B, C; *p* = 0.001), subsarcolemmal NOS1 localization (*p* = 0.001) was increased, whereas no change was found in astronaut D and reduced NOS1 signal intensities were found in astronaut E post- vs. pre-flight (Figure 3A). 

Eventually, a drop in sarcolemma NOS1 immunosignal found in the SDM astronaut was largely prevented in LDM astronauts (Figure 3A). With the exception of astronaut E, these findings support the notion that subsarcolemmal NOS1 expression likely reflects the positive muscle-type-specific effects induced by targeted inflight CM exercise protocols in three LDM crew members (astronauts B–D) in the deep calf soleus muscle.

Quantitative NOS1 biochemical analyses from soleus lysates of astronauts show that the amount of total NOS1 protein is changed in LDM B (trend) and LDM C (reduced) vs. SDM (no change) (see Figure 3B).

### 3.3. Nitro-DIGE Analysis of Astronaut Soleus Biosamples

**Figure 4:**
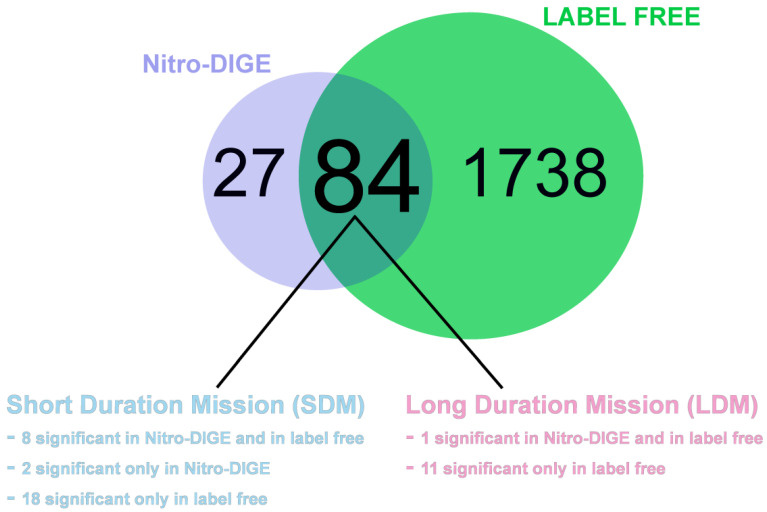
Venn diagram comparing the number of identified global proteins with posttranslational SNO modifications (total 111 proteins, Nitro-DIGE, violet circle) with total protein identified in label-free LC-MS/MS analysis (total 1822 proteins, LABEL FREE, green circle). A total of 84 were present both in Nitro-DIGE and label-free data (see overlapping area of circles), of which 28 or 12 were over-/under-nitrosylated in SDM or LDM. See also Supplementary Materials.

We then assessed the abundance of proteoforms provided by the Nitro-DIGE technology, which generates typical “nitro-profiles” from SNO proteins based on a modified biotin switch method [40] in which the labile SNO group is selectively reduced with ascorbate and labelled with CyDyes. Analyses were performed on pre- and post-flight soleus biopsy samples from a single non-exercise SDM astronaut (*n* = 1) and four LDM astronauts (*n* = 4) performing daily routine countermeasure protocols during their stay aboard the ISS. Changes in nitrosylation levels were assessed for each protein by normalizing the Nitro-DIGE level over the label-free LC-MS/MS abundance dataset previously published based on the same subjects [39]. Proteins showing both decreased abundance and nitrosylation or vice versa, after microgravity exposure, were not considered, as we would like to identify proteins that are over- or under-nitrosylated compared to under- or over-expressed total protein. A total of 1822 proteins were identified from SDM and LDM astronaut soleus muscle in the label-free analysis, whereas 111 spots were found nitrosylated in Nitro-DIGE, among which 84 were present in the label-free dataset (Figure 4). Of the 84 nitrosylated proteins identified, in SDM exposure, eight showed statistically significant differential expression in both Nitro-DIGE and label-free data, two were significantly over-nitrosylated in Nitro-DIGE only, and 18 showed differential abundance in label-free data, although nitrosylation changes were not statistically significant. In LDM exposure, only one protein was differentially nitrosylated before and after exposure in Nitro-DIGE and label-free data, whereas 11 were not differentially nitrosylated but were differentially abundant (Figure 4). The list of proteins differentially expressed in SDM and LDM, together with statistical analyses, protein accession number, and gene name, is available in Supplementary Tables S1 (SDM) and S2 (LDM). A representative “nitroprofile” of soleus muscle protein extracts from SDM and LDM obtained by Nitro-DIGE is available in Supplementary Figure S1.

**Figure 5:**
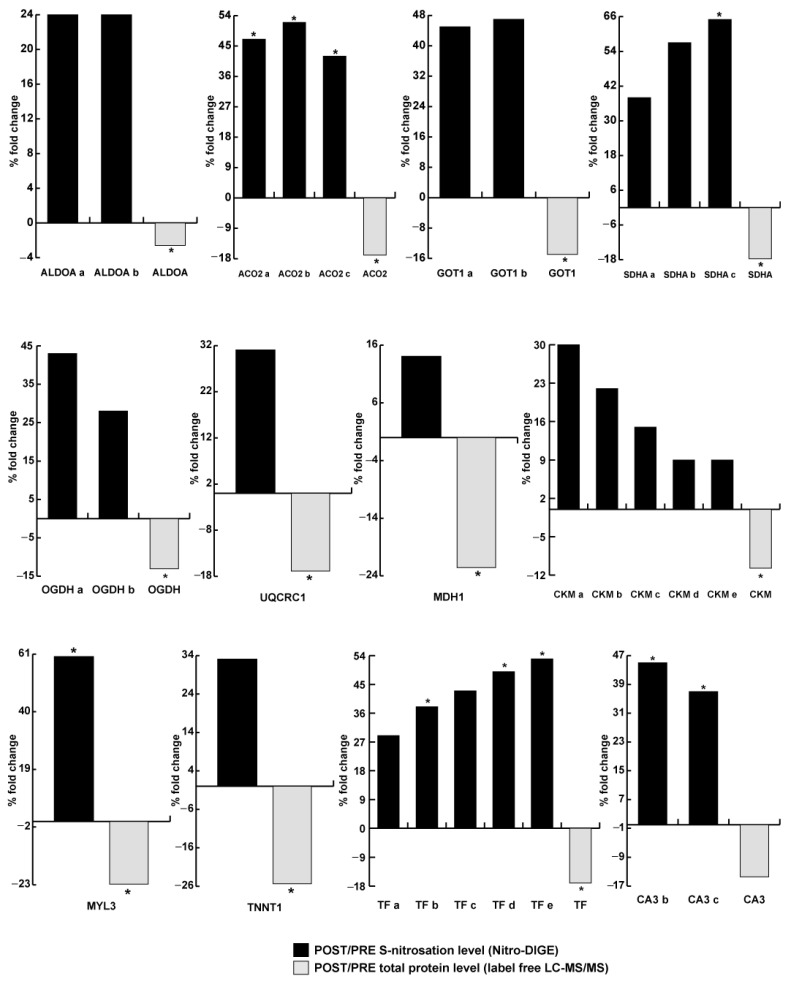
SDM Astronaut soleus nitrosoprofiles (s-nitrosylation) and abundance (total amount) of metabolic (glycolytic/TCA cycle), contractile muscle proteins (myosin light chain/troponins), and ion transporter serrotransferrin (TF). Total protein abundance (gray bars, label-free LC-MS/MS) and S-nitrosated (black bars, Nitro-DIGE) proteoforms (% fold change). Fructose-bisphosphate aldolase A (ALDOA), aconitate hydratase (ACO2), cytoplasmic aspartate aminotransferase (GOT1), succinate dehydrogenase [ubiquinone] flavoprotein subunit (SDHA), 2-oxoglutarate dehydrogenase complex component E2 (OGDH), cytochrome b-c1 complex subunit 1 (UQCRC1), cytoplasmic malate dehydrogenase (MDH1), creatine kinase M-type (CKM), myosin light chain 3 (MYL3), slow skeletal muscle troponin T (TNNT1), serotransferrin (TF), and carbonic anhydrase 3 (CA3) in SDM post vs. pre. (* = significant difference; POST vs. PRE SDM, Student’s *t*-test and FDR, *n* = 1).

Figure 5 shows the correlation between level of nitrosylation and protein abundance in the SDM astronaut, and shows a ‘statistically significant or only trend’ increase in the level of nitrosylation despite a significant decrease in the total protein abundance of several proteoforms involved in energy metabolism, transport, stress response, and muscle contraction. Specifically, in the glycolytic pathway, fructose-bisphosphate aldolase A proteoforms (ALDOA a, b) were over-nitrosylated, while in the TCA cycle, OXPHOS, and malate/aspartate shuttle, increased nitrosylation was observed in aconitate hydratase proteoforms (ACO2 a, b, c), cytoplasmic aspartate aminotransferase proteoforms (GOT1 a, b), succinate dehydrogenase [ubiquinone] flavoprotein subunit proteoforms (SDHA a, b, c), 2-oxoglutarate dehydrogenase complex component E2 proteoforms (OGDH a, b), cytochrome b-c1 complex subunit 1 (UQCRC1), cytoplasmic malate dehydrogenase (MDH1), and creatine kinase M-type proteoforms (CKM a, b, c, d, e). Among the contractile proteins, myosin light chain 3 (MYL3) and slow skeletal muscle troponin T (TNNT1) were over-nitrosylated as serum transferrin proteoforms (TF a, b, c, d, e) and carbonic anhydrase 3 proteoforms (CA b, c).

**Figure 6:**
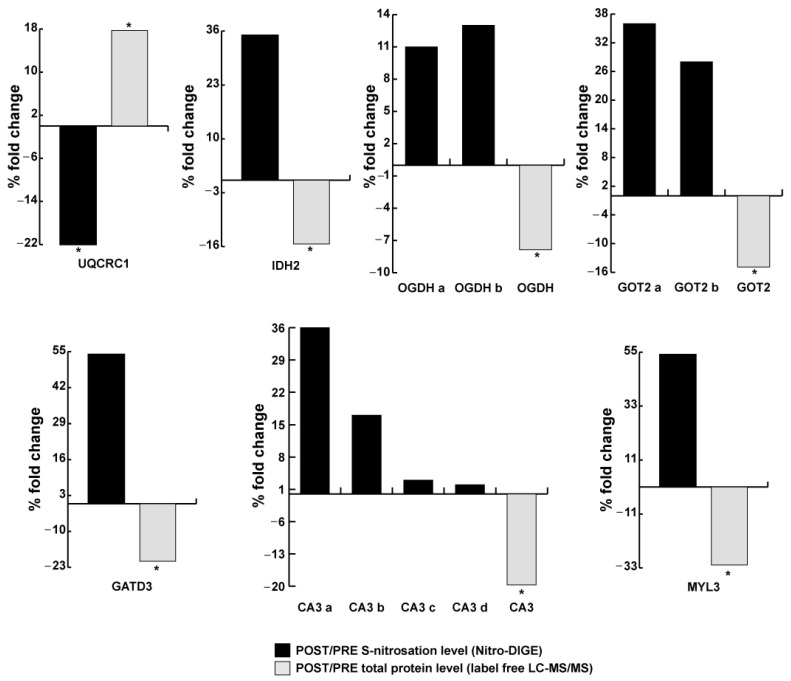
LDM astronaut soleus nitrosoprofiles (s-nitrosylation) and abundance (total amount) of mitochondria-related (respiratory chain) and structural (MYL3) proteins. Total protein abundance (gray bars, label-free LC-MS/MS) and S-nitrosated (black bars, Nitro-DIGE) level variations (% fold change) of cytochrome b-c1 complex subunit 1 (UQCRC1), isocitrate dehydrogenase [NADP] (IDH2), 2-oxoglutarate dehydrogenase complex component E2 (OGDH), mitochondrial aspartate aminotransferase (GOT2), glutamine amidotransferase-like class 1 domain-containing protein 3 (GATD3), carbonic anhydrase 3 (CA3), and myosin light chain 3 (MYL3) in LDM post vs. pre. (* = significant difference; POST vs. PRE LDM, paired Student’s *t*-test and FDR, *n* = 4).

Figure 6 presents the relation between level of nitrosylation and protein abundance in LDM astronauts. A significant decrease in nitrosylation despite an increase in protein abundance was observed for UQCRC1. At variance, for isocitrate dehydrogenase [NADP] (IDH2), proteoforms of OGDH, mitochondrial aspartate aminotransferase proteoforms (GOT2 a, b), glutamine amidotransferase-like class 1 domain-containing protein 3 (GATD3), proteoforms of CA3, and MYL3, the total abundance was decreased, while levels of nitrosylation were increased, although not significantly changed. 

**Figure 7:**
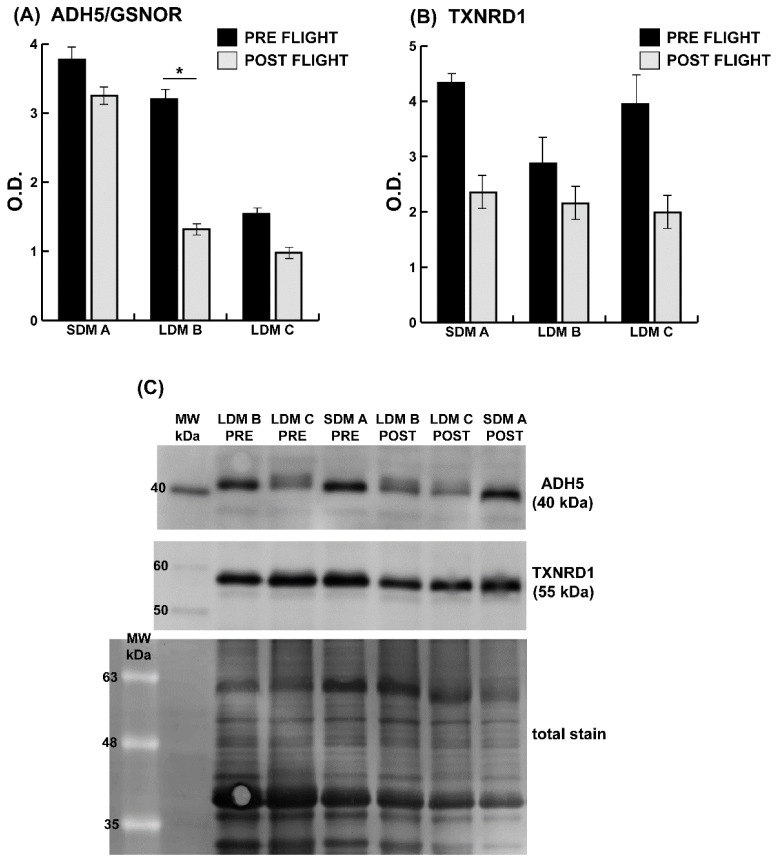
Immunoblots of cytosolic denitrosylation enzymes in SDM vs. LDM astronaut soleus. Representative histograms (means ± SD) showing protein abundance of (**A**) alcohol dehydrogenase 5/S-nitrosoglutathione reductase (ADH5/GSNOR) and (**B**) thioredoxin reductase 1 (TXNRD1) in preflight (black bars) vs. postflight (grey bars) in one (SDM A) astronaut, and two (LDM-B/-C) astronauts. (**C**) Representative immunoblot images from pre- vs. postflight SDM-A and LDM-B/-C. Data were normalized against the total amount of loaded proteins stained with Sypro Ruby. O.D. = optical density; * significant difference; Student’s *t*-test, *n* = 2, *p* < 0.05. Full-length images are available in the Supplementary Materials.

Figure 7 depicts the levels of molecules known to control nitrosative stress, such as alcohol dehydrogenase 5/S-nitrosoglutathione reductase (ADH5/GSNOR) and thioredoxin reductase 1 (TXNRD1), as assessed by immunoblotting in one SDM and two LDM astronauts. Results indicate that both ADH5 and TXNRD1 levels were decreased in all subjects, but only ADH5 in LDM (Figure 7B) was significant. 

## 4. Discussion

The present work reports that in contrast to the SDM condition in spaceflight (acute microgravity impact), LDM astronauts (chronic µG impact) showed that (i) sarcolemmal NOS-type 1 dislocation in soleus myofibers was not evident shortly after return from spaceflight, and (ii) a restricted set of functional muscle proteins (structural, metabolic, mitochondrial) were over-nitrosylated (s-nitrosylated vs. total protein abundance) after spaceflight compared to pre-flight controls. This work presents a novel dataset of s-nitrosylated skeletal muscle proteins of astronaut bio-samples (muscle biopsy). It aimed to gain insight into organ-based oxidative homeostasis in spaceflight [43] and to complement interdisciplinary space-related space-omics research databases on various space-flown biological organisms [44,45,46], development of countermeasures in personalized space medicine [47], and recent human space-omics reports [48,49]. Sarcolemmal NOS1 expression and protein s-nitrosylation in skeletal muscle are further keys to better understanding the molecular networks associated to the acute or chronic nitrosative stress management to defend skeletal muscle dysfunctions and impaired performance control in human spaceflight. 

Altered sarcolemmal NOS1 expression is an oxidative stress-dependent early event and valuable cell marker linked not only to muscle activity/inactivity but also to various muscle pathologies [50]. Functionally distinct neuronal NOS (nNOS/NOS1) and its known splice variants (nNOSµ, nNOSß) are localized in muscle fibers to the sarcolemma (nNOS via PDZ domains of syntrophin to dystrophin glycoprotein complex), cis-Golgi complex (nNOSß), and cytoplasm (Balke et al., 2019), thus reflecting a complex interplay in various subsystems via NO/NOS signaling in normal muscle functions [51]. A fine regulation of myofiber function and homeostasis is apparently lost when nNOS (NOS1) dislocates from the sarcolemma to the cyto(sarco)solic compartment following disuse conditions [50]. Similar immuno-patterns of NOS1 were found in the apparently hypoactive or even passive soleus muscles of the SDM astronaut in the absence of a prescribed exercise training protocol because of the relatively short duration of space travel (1.5 weeks SDM vs. 24 weeks LDM) combined with a minimal risk for very low to moderate disuse atrophy with little impact on muscle health condition during short spaceflight and recovery thereafter. Increased s-nitrosylation of cytosolic, contractile proteins, and ion transporters was detectable in the SDM astronaut (Figure 5) despite similar NOS1 abundance in SDM pre- vs. post-flight (Figure 3B) measures, suggesting an interplay between alternative myofiber NO sources (NOS1, inducible NOS2) expressed in human skeletal muscle [38].

Reduced subsarcolemmal NOS1 is also an early event driving disuse-induced muscle atrophy in rats and short-term bed rest [50], as well as in long-term bed rest [38] and in space-flown mice [37], and thus is considered a “non-exercise” reference to the NOS1 immuno-expression levels found in our study of LDM astronauts (B to E) who performed up to 2.5 h of regular daily inflight exercise sessions during their 6-month missions onboard the ISS. All four LDM astronauts studied in this work showed preservation of sarcolemmal NOS1 immuno-reactivity around their soleus muscle myofibers post- vs. pre-flight, which is plausibly due to their routine inflight countermeasure prescriptions that included, in particular, daily inflight exercise with the advanced resistive exercise device (aRED) [52] as an integral part of the routine inflight countermeasure portfolio throughout their six-month missions onboard the ISS [53]. 

In the SDM astronaut, we found nitrosylation of (1) a nodal protein of the glycolytic flux, (2) specific proteins in TCA cycle and respiratory chain, (3) over-nitrosylation of proteins involved in muscle contraction, and (4) CK together with decrease in antioxidant protection as well as nitrosylation of TF that can promote ferroptosis (intracellular ion-dependent cell death [54]) as signs of microgravity muscle maladaptation. 

In LDM astronauts, nitroso-profiles of some muscle proteins reflected a recovery of the glycolytic (ALDO) and energy flux (CKM), and of several enzymes of the TCA cycle (ACO2 and MDH1), while a recovery of mitochondrial activity was observed with no differential change in protein abundance and undetectable nitrosylation for SDHA. However, a significant increase in protein abundance was observed for UQCRC1 coupled with a decrease in nitrosylation. A decreased protein level with a tendency to retain an over-nitrosylated state was observed for mitochondrial GATD3, IDH2, GOT2, and OGDH. The same occurred with MYL3, whereas TNNT1 and TF were normalized as signs of muscle adaptation to microgravity. Notably, independent of the mission duration in space, levels of enzymes mediating denitrosylation of ADH5/GSNOR and TXNRD1 [41] were detectable as unmistakable signs of impaired redox homeostasis and reduced protection from nitrosative stress in muscle. Immunoblotting indicated decreased levels of NOS1 in muscle tissue that likely reflected a protective mechanism. Over-nitrosylation of enzymes in the TCA cycle as well as proteins in the electron transport chain can hinder the efficiency of oxidative phosphorylation and subsequently ATP synthesis [55]. We can hypothesize that over-nitrosylation of contractile proteins may reflect a protective mechanism to preserve muscle contraction homeostasis, reducing Ca^2+^ sensitivity and ATPase activity [56,57].

The use of Nitro-DIGE technology represents a reliable methodological tool that allowed us to identify variable expression patterns (loss vs. gain) detectable by individual nitrosoprofiles of functional proteins (structural/contractile, energy metabolism, transport, stress response, mitochondria) as SNO signatures from astronauts (short vs. long duration). Functional protein nitroso-profiles included members of primary contractile proteins, such as myosin light chains (MYL-3), Ca^2+^-binding proteins, which modulate force transduction and cross-bridge kinetics, which were found over-nitrosylated in both SDM and LDM subjects (Supplementary Tables S1 and S2). Essential fast myosin light chains (MYL-3/MLC-3) belong to key contractile proteins like Myosin heavy chains (MyHCs), troponins (TnTs 1-3))/tropomyosins (TPM1-3), and α-actins (ACTA1) from skeletal muscle and are considered hallmarks of fiber type transition in the muscle aging process (sarcopenia) that leads to atrophy and frailty [58]. Earlier work on muscle contractile function with exercise also showed loss of peak forces in skinned myofibers from a slow soleus muscle after 17 d spaceflight [59] and increased MLC-3 subunit in soleus and extensor digitorum longus (EDL) muscles. More interestingly, protein content in MYL-3 was up-regulated in competing runners vs. recreational runners, suggesting MLC compositional changes in extreme loading conditions (endurance training) [59]. MYL-3 decreased in aged muscle, suggesting weakness in aging population [60]. In this respect, microgravity adaptation of inactive vs. active astronaut soleus muscle is linked to altered SNO patterns of contractile muscle proteins, and presumably also linked to myofiber type shifts (slow to fast). 

We also found elevated SNO levels of carbonic anhydrases (CA), CA3b and CA3c, in both LDM and SDM astronauts. Cytosolic muscle proteins like CAs mediate reversible hydration of carbon dioxide. Among others, CAs are frequently found in the human proteome, including muscle [61]. Moreover, CA3 is enriched in slow fibers and an ideal marker for fiber shift in muscle adaptation [62], aging [63], and also in disused vastus lateralis muscles in bed rest, a human immobilization study as an analog to extended spaceflight [42]. These findings show that altered nitrosylation products are to be investigated in various cellular subsystems following skeletal muscle adaptation linked to various atrophy conditions including disuse, aging, and disease, but also spaceflight, with as-yet un-estimated consequences for dysfunctional human skeletal muscle on Earth and in space. 

Aberrant s-nitrosylation is a hallmark of muscle wasting and neuromuscular dysfunction of human skeletal muscle [64]. Low levels of ADH/GSNOR are detected in muscular dystrophies, muscle atrophy, aging, amyotrophic lateral sclerosis (ALS), and neurodegenerative diseases, thus suggesting a key role in overall muscle homeostasis. Moreover, NOS1/GSNOR contributes to muscle differentiation and homeostasis [41]. We were able to identify decreased levels of the denitrosylation enzymes ADH5/GSNOR [41] and of the cytosolic selenoprotein TRXRD1, both linked to the transcription factor Nrf2, a potent regulator essential to the maintenance of redox homeostasis [65]. The level of TRXRD1 showed a trend in both SDM (non-exercise) and LDM (inflight exercise) conditions, and ADH5/GSNOR was significantly decreased in the LDM-B astronaut post- vs. pre-flight, suggesting a risk of insufficient mitigation effects of currently available inflight exercise training protocols, particularly regarding those affecting redox homeostasis and RONS-linked skeletal muscle properties in human muscle on Earth [9] and concerning microgravity-induced muscle hypoactivity/disuse in space.

NO is principally produced by NOS1 in contracting muscle fibers by the NADPH oxidase enzymes, NOX2 (plasma membrane) and NOX4 (intracellular membranes), but also by mitochondria [66,67]. Mitochondrial signaling also contributes to disuse muscle atrophy [68], and is common to heart and skeletal muscle pathologies, including sarcopenia [69]. We found robust recovery signs of mitochondrial activity in LDM astronaut bio-samples. Mitochondrial homeostasis and redox status appear to be highly challenged in spaceflight, as previously analyzed in hair-samples from LDM astronauts (collected pre-, in-, and post-flight) showing mitochondrial damage (reduced mtDNA/nDNA) together with other redox signal genes involved in oxidative stress observed post-flight [7]. 

In the skeletal muscle of astronauts, we found mitochondrial aconitate hydratase enzyme isoforms (i.e., ACO2a-c) as part of the TCA cycle highly nitrosylated in the SDM astronaut, while other mitochondrial enzymes, such as aspartate aminotransferases (GOT2), but also isocitrate dehydrogenase (NADP) (IDH2), were nitrosylated following LDM spaceflight, suggesting mitochondrial enzymes are likely targets for various mitochondrial functions related to spaceflight impact [70]. 

Previous other work with healthy subjects found elevated citrate as a potential stress response to preserve redox status of the cell during intense exercise [71] and aconitase post-translational modification as hallmarks in redox signaling and metabolism of ROS [72], further suggesting nitrosylation of mitochondrial proteins as another post-translational modification in skeletal muscle metabolism during spaceflight. Aconitase (ACOs) and oxoglutarate dehydrogenase (OGDH) inhibition via NO in rat soleus skeletal muscle strips in vitro suggests an impact of NO on catalytic enzyme functions in skeletal metabolism [73]. Mammalian GATD3 is a mitochondrial matrix enzyme with bacterial provenance that functions as a deglycase and interacts with the mitochondrial translation machinery to stabilize mitochondrial integrity involved in mitochondrial glycation status and thus dynamics [74]. It has been shown to be highly nitrosylated (54%-fold change) in LDM samples. Data from SDHA proteomic changes during bed rest [75,76], as well as conventional SDH histochemistry on muscle cross-sections after 11 days of spaceflight [77], support our results regarding the possible reduced metabolic support via mitochondria under the non-exercise SDM condition studied in this work.

Skeletal muscle function is linked to a well-balanced and coordinated redox production and detoxification system for physiological function [78]. Oxidative stresses are closely linked to mechanical loading stress for maximal force generation [27,79]. Hyper-nitrosylated RyR calcium release channels are leaky in dystrophic muscle [80]. Redox balance (cellular redox state) fine-tunes muscle hypertrophy and NO exerts a negative effect on Akt/mTORc1 function (hypertrophy), possibly to keep hypertrophy in check [81]. 

Nitrates and nitrites can serve as food sources for health benefit [82]. NO synthesis in tissue can occur through reduction of nitrate to nitrite, and nitrite can be subsequently reduced to NO as a systemic reservoir for NO production in muscle; thus, a physiological benefit with a provision of nitrite from dietary sources appears likely. However apart from physiological roles, nitrate and nitrite aspects of toxicity related to excess uptake on circulation and muscle remain to be properly assessed [83], in particular with respect to space-related nutritional countermeasures.

Increased inflight oxidative stress has previously been suggested with regard to human spaceflight [84]. To date, antioxidant/anti-inflammatory cocktails have been tested against disuse-induced muscle deconditioning in bed rest [12,42,85] based on nutritional genomics and nutrigenetics to minimize ROS damaging effects during manned spaceflight; such cocktails are currently being assessed for future space travelers and astronauts (in particular, those involving Vitamins A, C, E, and D, selenium, resveratrol, and others) [11]. We previously investigated muscular SNO protein signatures by resistive and vibration exercise in bed rest as an analogue to spaceflight [11], the results of which suggest a high potential for exercise to offset nitrosative stress and damage in chronic disuse conditions on Earth. Exercise-induced changes (endurance, high intensity, resistive) [8] in skeletal muscle antioxidant enzymes have been reported. Likewise, a therapeutic potential of denitrosylation to defend against nitrosative stress in human disease was also recently suggested [9]. For mission success and safety of future spaceflight, disrupted redox homeostasis in LDM astronauts’ skeletal muscle, as shown in this work, calls for the development of multimodal inflight countermeasures, with exercise and nutritional supplementation, for improved nitrosative stress management in order to minimize disrupted redox homeostasis of skeletal muscle.

Study limitations exist, including (1) the small number of participants of the study (which is an inherent limitation factor for all space-related research); (2) limited numbers of biopsies, highlighted by the fact that only one (*n* = 1) “non-exercise” SDM crewmember (9 days on ISS) was available for this study, which limits statistical strength; and (3) the fact that regular and rigorous inflight CM exercise protocols (up to 2.5 h daily per week) have already prescribed for LDM astronauts onboard the ISS for many years by the relevant Space Agencies [86,87]. Due to ethical reasons, biopsies from “non-exercise” LDM crew members on ISS (inflight negative controls) or inflight biopsies were not available, for obvious reasons. Nevertheless, small muscle biopsy samples obtained from a unique population of astronauts help to address proof of concept/hypotheses, for instance, with hypotheses centered on microgravity-induced molecular adaptation processes (reflected, for example, by canonical and other muscle-specific biological pathways otherwise being masked by gravity on Earth [39] and with potential benefit for better health management of people on Earth).

## 5. Conclusions

Aberrant levels of SNO-modified proteins of different subsystems investigated in the slow-type soleus muscle are potential triggers for muscle dysfunctions known from health risk assessments in human spaceflight. The present work showed that sarcolemmal NOS1 dislocation can be at least partly mitigated in actively contracting myofibers of the postural deep calf soleus muscle, suggesting efficacy of the currently available inflight exercise protocol onboard the ISS. However, over-nitrosylation of key functional muscle proteins (structural, metabolic, mitochondria complex) and decreased levels of the two major denitrosylase enzymes, TXNRD1 and GSNOR, are hallmarks of impaired redox homeostasis in astronaut skeletal muscle, independent of the time of microgravity exposure (SDM acute vs. LDM chronic). The complex patterns of under-/over-nitrosylation of specific muscle protein still remain to be analyzed for other muscle groups in order to establish whether peculiar SNO signatures reflect a protective or detrimental mechanism in microgravity adaptation of the astronauts’ musculature. This should, however, be considered for the development of next-generation inflight countermeasures targeted to alleviate imbalance in oxidative homeostasis, evidenced by aberrant muscular nitrosoprofiles reported by the present work. Future inflight countermeasures, but also recovery protocols thereafter, should also include nutritional supplementation, for example, by selenoproteins or other antioxidant nutrients to minimize muscular nitrosative stress with impact on mission crew health and performance control in future human deep space exploration missions. 

## Data Availability

All human datasets analyzed during the current study are not publicly available due to privacy reasons but are available from the corresponding author upon reasonable request.

## Supplementary Materials

The following supporting information can be downloaded at: https://www.mdpi.com/article/10.3390/antiox13040432/s1, Figure S1: Nitro-DIGE gel images of SOL muscle protein extracts from one short-duration (9 days on ISS, without inflight exercise; acute µG exposure) and four long-duration (>6 month or more on ISS, with routine inflight exercise; chronic µG exposure) mission astronauts (SDM, left panel; LDM, right panel); Table S1: List of identified proteins in Short Duration Mission (SDM) group by NITRO-DIGE and label-free LC-MS/MS analysis; Table S2: List of identified proteins in Long Duration Mission (LDM) group by NITRO-DIGE and label-free LC-MS/MS analysis.

## Abbreviations

ADH5	alcohol dehydrogenase 5
ALDOA	Fructose-bisphosphate aldolase A
ACO2	aconitate hydratase proteoforms (a, b or c)
ARED	advanced resistive exercise device (on ISS)
BDC	Baseline data collection (on the ground, pre/postflight)
CA3	carbonic anhydrase 3
CEVIS	cycle ergometer with vibration isolation and stabilization (on ISS)
CKM	creatine kinase M-type
CM	countermeasure (physical exercise intervention)
DLR	Deutsches Zentrum für Luft- und Raumfahrt (German Aerospace Agency)
EAC	European Astronaut Center (Cologne)
ESA	European Space Agency
FDR	False discovery rate (statistics)
G	gravity vector (1G Earth/µG Space)
GATD3	glutamine amidotransferase-like class 1 domain-containing protein 3
GOT1	cytoplasmic aspartate aminotransferase
GOT2	mitochondrial aspartate aminotransferase
GSNOR	S-nitrosoglutathione reductase
IDH2	isocitrate dehydrogenase [NADP]
IPG	immobilized pH gradient
ISS	International Space Station
JAXA	Japanese Space Agency
LDM	long-duration mission (>180 days)
MDS	Mice drawer system (habitat module on ISS)
MedOPs	Medical operational team
MDH1	cytoplasmic malate dehydrogenase
MYL3	myosin light chain 3
M.O.M.	mouse-on-mouse (immunocytochemical blocking agent)
NASA	National Aeronautics and Space Administration
Nitro-DIGE	S-Nitrosated proteins identified by 2-D CyDye-Maleimide
NO	nitric oxide
NOS	NO synthase
O.D.	optical density
OGDH	2-oxoglutarate dehydrogenase complex component E2
R+0/1	return day (landing day on Earth)
ROS	reactive oxidative species
RNS	reactive nitrogen species
RONS	reactive oxidative and nitrosative species
SDHA	succinate dehydrogenase [ubiquinone] flavoprotein subunit
SDM	short-duration mission (11 days spaceflight)
T2	treadmill on ISS
TF	serotransferrin
TNNT1	slow skeletal muscle troponin T
TXNRD1	thioredoxin reductase 1
UQCRC1	cytochrome b-c1 complex subunit 1
USOS	United States orbital system
µG	microgravity
