# Supplementary material for: Nitrosative Stress in Astronaut Skeletal Muscle in Spaceflight
Source: Antioxidants (Basel). 2024 Apr 2;13(4):432. doi: 10.3390/antiox13040432 (PMC11047620; doi:10.3390/antiox13040432)
Supplement: Supplementary file 1 [file antioxidants-13-00432-s001.zip › antioxidants-2892664-supplementary.pdf]

Supplementary Materials

Figure S1. Nitro-DIGE gel images of SOLEUS muscle extracts from SDM / LDM Astronauts

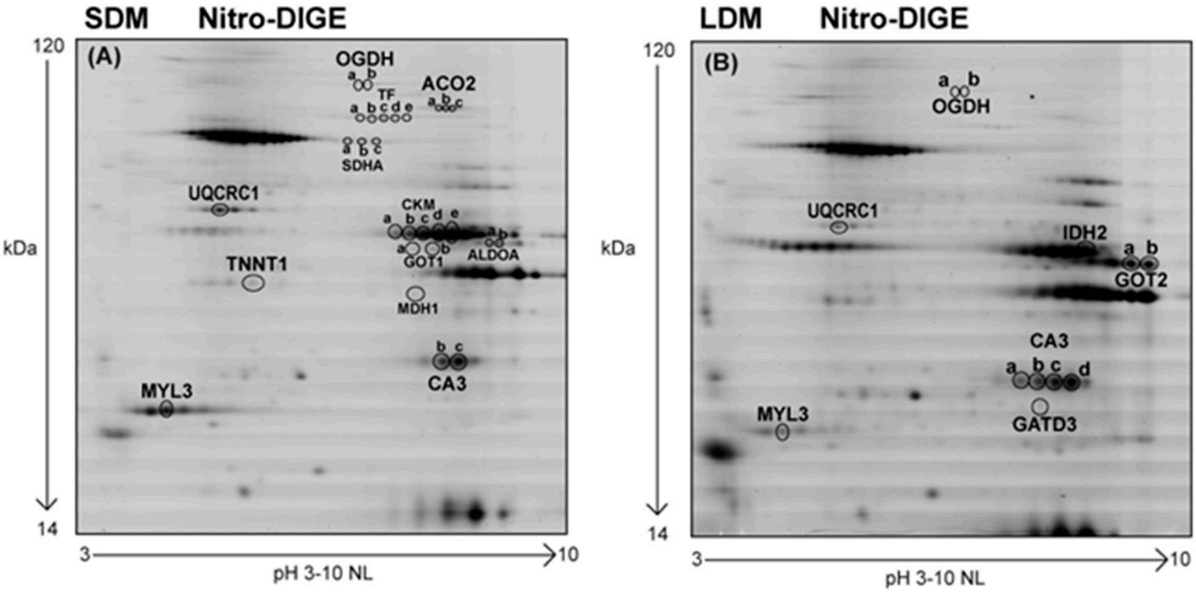

Figure S1. Nitro-DIGE gel images of SOL muscle protein extracts from one short-duration (9 days on ISS, without inflight exercise; acute μG exposure) and four long-duration (> 6 month or more on ISS, with routine inflight exercise; chronic μG exposure) mission astronauts (SDM, left panel; LDM, right panel). Protein extracts (20 μg for each sample) were run in duplicate using 24 cm, 3–10 non-linear immobilized pH-gradient (IPG) strips. Isoelectric focusing was performed on an IPGphor electrophoresis unit (GE Healthcare) using a gradient ranging from 200 to 8000 V, reaching a total of 75,000 Vh. Focused proteins were reduced and alkylated prior to second dimension electrophoretic run. Second dimension was performed on 20x25 cm2, 12% T, 2.5% C constant concentration polyacrylamide gels at 20 C and 15 mA using the Ettan Dalt II system (GE Healthcare). Images of CyDye-labeled gels were acquired using a Typhoon 9200 Imager (GE Healthcare), and image analysis was performed using DeCyder software (version 6.5, GE Healthcare). Gel images show nitrosylated spots. Changes in nitrosylation levels were assessed for each protein by normalizing the Nitro-DIGE level to the previously published label-free LC-MS/MS abundance data set performed on the same subjects. **(Left panel)** Among 111 nitrosylated spots, 28 were identified as differentially nitrosylated in pre *vs* postflight SDM. Proteoforms of fructose-bisphosphate aldolase A (ALDOA), proteoforms of aconitate hydratase (ACO2), proteoforms of cytoplasmic aspartate aminotransferase (GOT1), proteoforms of succinate dehydrogenase [ubiquinone] flavoprotein subunit (SDHA), proteoforms of 2-oxoglutarate dehydrogenase complex component E2 (OGDH), cytochrome b-c1 complex subunit 1 (UQCRC1), cytoplasmic malate dehydrogenase (MDH1), proteoforms of creatine kinase M-type (CKM), myosin light chain 3 (MYL3), slow skeletal muscle troponin T (TNNT1), proteoforms of serotransferrin (TF), and protoeforms of carbonic anhydrase 3 (CA3). **(Right panel)** Among 111 nitrosylated spots, 12 were identified as differentially nitrosylated in pre *vs* postflight LDM. UQCRC1, isocitrate dehydrogenase [NADP] (IDH2), proteoforms of OGDH, proteoforms of mitochondrial aspartate aminotransferase (GOT2), glutamine amidotransferase-like class 1 domain-containing protein 3 (GATD3), proteoforms of CA3, and MYL3.

Table S1. List of identified proteins in Short Duration Mission (SDM) group by NITRO-DIGE and label-free LC-MS/MS analysis.

Table S1. List of identified proteins in Short Duration Mission (SDM) group by NITRO-DIGE and label-free LC-MS/MS analysis.

NITRO DIGE
LABEL FREE

			NITRO DIGE		LABEL FREE	
Protein IDs	Gene names	Protein Name	T-test SDM	% fold change POST/PRE SDM	Student's T-test POST/PRE SDM	% fold change POST/PRE SDM
P04075	ALDOA a	Fructose-bisphosphate aldolase A	0,381	24	0,0325902	-2,58
P04075	ALDOA b	Fructose-bisphosphate aldolase A	0,408	24		
Q99798	ACO2 a	Aconitate hydratase, mitochondrial	0,0135	47	0,0245542	-16,91
Q99798	ACO2 b	Aconitate hydratase, mitochondrial	4,22E-03	52		
Q99798	ACO2 c	Aconitate hydratase, mitochondrial	1,75E-03	42		
P17174	GOT1 a	Aspartate aminotransferase, cytoplasmic	0,448	45	0,0422065	-14,90
P17174	GOT1 b	Aspartate aminotransferase, cytoplasmic	0,22	47		
P31040	SDHA a	Succinate dehydrogenase [ubiquinone] flavoprotein subunit, mitochondrial	0,131	38	0,0294336	-17,67
P31040	SDHA b	Succinate dehydrogenase [ubiquinone] flavoprotein subunit, mitochondrial	0,0504	57		
P31040	SDHA c	Succinate dehydrogenase [ubiquinone] flavoprotein subunit, mitochondrial	0,0231	65		
Q02218	OGDH a	2-oxoglutarate dehydrogenase complex component E1	0,126	43	0,0128131	-13,04
Q02218	OGDH b	2-oxoglutarate dehydrogenase complex component E2	0,126	28		
P31930	UQCRC1	Cytochrome b-c1 complex subunit 1, mitochondrial	0,0852	31	0,0314967	-16,88
P40925	MDH1	Malate dehydrogenase, cytoplasmic	0,793	14	0,0307787	-22,56
P06732	CKM a	Creatine kinase M-type	0,12	30	0,0369019	-10,73
P06732	CKM b	Creatine kinase M-type	0,0992	22		
P06732	CKM c	Creatine kinase M-type	0,16	15		
P06732	CKM d	Creatine kinase M-type	0,5	9		
P06732	CKM e	Creatine kinase M-type	0,458	9		
P08590	MYL3	Myosin light chain 3	6,22E-03	60	0,0314967	-22,74
P13805	TNNT1	Troponin T, slow skeletal muscle	0,0817	33	0,0129625	-25,31
P02787	TF a	Serotransferrin	0,093	29	0,0226543	-17,04
P02787	TF b	Serotransferrin	8,27E-03	38	0,0226543	-17,04
P02787	TF c	Serotransferrin	0,0599	43		
P02787	TF d	Serotransferrin	0,0402	49		
P02787	TF e	Serotransferrin	0,0248	53		
P07451	CA3 b	Carbonic anhydrase 3	0,0284	45	0,120365	-14,44
P07451	CA3 c	Carbonic anhydrase 3	0,0204	37		

Table S2. List of identified proteins in Long Duration Mission (LDM) group by NITRO-DIGE and label-free LC-MS/MS analysis.

Table S2. List of identified proteins in Long Duration Mission (LDM) group by NITRO-DIGE and label-free LC-MS/MS analysis.

NITRO DIGE
LABEL FREE

			NITRO DIGE		LABEL FREE	
Protein IDs	Gene names	Protein Name	Paired T-test LDM	% fold change LDM POST/PRE	Paired T-test POST/PRE LDM	% fold change POST/PRE LDM
P31930	UQCRC1	Cytochrome b-c1 complex subunit 1, mitochondrial	0,007040	-22	0,0073591	17,74
P48735	IDH2	Isocitrate dehydrogenase [NADP], mitochondrial	0,423	35	0,000236415	-15,37
Q02218	OGDH a	2-oxoglutarate dehydrogenase complex component E1	0,977	11	0,0193319	-7,86
Q02218	OGDH b	2-oxoglutarate dehydrogenase complex component E2	0,958	13		
P00505	GOT2 a	Aspartate aminotransferase, mitochondrial	0,726	36	0,000243915	-14,91
P00505	GOT2 b	Aspartate aminotransferase, mitochondrial	0,812	28		
P0DPI2	GATD3	Glutamine amidotransferase-like class 1 domain-containing protein 3, mitochondrial	0,109	54	0,000715169	-20,77
P07451	CA3 a	Carbonic anhydrase 3	0,284	36	0,000246763	-19,71
P07451	CA3 b	Carbonic anhydrase 3	0,297	17		
P07451	CA3 c	Carbonic anhydrase 3	0,699	3		
P07451	CA3 d	Carbonic anhydrase 3	0,928	2		
P08590	MYL3	Myosin light chain 3	0,368	54	0,000321484	-31,81
